# The effects of propofol anaesthesia on molecular-enriched networks during resting-state and naturalistic listening

**DOI:** 10.1016/j.neuroimage.2023.120018

**Published:** 2023-05-01

**Authors:** Timothy Lawn, Daniel Martins, Owen O'Daly, Steve Williams, Matthew Howard, Ottavia Dipasquale

**Affiliations:** Department of Neuroimaging, Institute of Psychiatry, Psychology and Neuroscience, King's college London, London, UK

**Keywords:** Anaesthesia, Consciousness, fMRI, Molecular, Receptor, Naturalistic

## Abstract

Placing a patient in a state of anaesthesia is crucial for modern surgical practice. However, the mechanisms by which anaesthetic drugs, such as propofol, impart their effects on consciousness remain poorly understood. Propofol potentiates GABAergic transmission, which purportedly has direct actions on cortex as well as indirect actions via ascending neuromodulatory systems. Functional imaging studies to date have been limited in their ability to unravel how these effects on neurotransmission impact the system-level dynamics of the brain. Here, we leveraged advances in multi-modal imaging, Receptor-Enriched Analysis of functional Connectivity by Targets (REACT), to investigate how different levels of propofol-induced sedation alter neurotransmission-related functional connectivity (FC), both at rest and when individuals are exposed to naturalistic auditory stimulation. Propofol increased GABA-A- and noradrenaline transporter-enriched FC within occipital and somatosensory regions respectively. Additionally, during auditory stimulation, the network related to the dopamine transporter showed reduced FC within bilateral regions of temporal and mid/posterior cingulate cortices, with the right temporal cluster showing an interaction between auditory stimulation and level of consciousness. In bringing together these micro- and macro-scale systems, we provide support for both direct GABAergic and indirect noradrenergic and dopaminergic-related network changes under propofol sedation. Further, we delineate a cognition-related reconfiguration of the dopaminergic network, highlighting the utility of REACT to explore the molecular substrates of consciousness and cognition.

## Highlights

• Consciousness emerges from complex interactions across organisational hierarchies.
• The dopaminergic network shows cognition-related modulation under anaesthesia.
• GABAergic and noradrenergic circuits undergo functional changes during sedation.
• Multimodal imaging offers new insight into the complex mechanisms of consciousness.

## 1 Introduction

The ability of anaesthesia to transiently and reversibly disrupt conscious experience has both revolutionised modern surgical practice as well as provided a unique opportunity to link consciousness to its neurobiological substrates. However, the mechanisms through which this altered state of consciousness emerges are far from fully elucidated, in part due to the multiplicity of contributing systems which interact at multiple levels (Alkire et al., 2008; Brown et al., 2010; Franks, 2008). A thorough characterisation of these different systems supporting consciousness may also offer novel therapeutic targets for disorders of consciousness, for which we remain largely bereft of meaningful treatments. As such, mechanistic investigation of anaesthetic agents and their pharmacodynamic effects may provide fundamental insights into the brain, with applications in both health and disease.

Much neuroimaging work investigating anaesthesia to date has leveraged the powerful analytic approaches applied to blood-oxygen level dependant (BOLD) signal fluctuations measured with functional magnetic resonance imaging (fMRI) whilst participants are at rest. Far more than a baseline, this resting activity reflects inherent functional organisation of the brain as well as personal mentation (Binder et al., 1999; Buckner, 2012; Stark and Squire, 2001). Anaesthesia seems to preferentially perturb certain domains of brain function, having been largely described to reduce within and between higher-level network connectivity, while preserving sensory processing within lower-level primary sensory cortices (Adapa et al., 2014; Boveroux et al., 2010; Craig et al., 2021; Gili et al., 2013; Gómez et al., 2013; Guldenmund et al., 2013; Liu et al., 2017; Mhuircheartaigh et al., 2010; Monti et al., 2013; Naci et al., 2018; Nir et al., 2019; Qiu et al., 2017; Schröter et al., 2012; Schrouff et al., 2011; Spindler et al., 2021; Stamatakis et al., 2010; Tang and Ramani, 2016). As such, studies considering only the resting state are limited in their ability to comprehensively investigate anaesthesia's effects on the brain. Naturalistic stimuli offer a powerful tool to engage ecologically meaningful sensory and higher-level cognitive processes that do not also necessitate responses (Finn, 2021). Therefore, they are well suited to examine how the neural substrates of perception and cognition persist or are extinguished under anaesthesia.

The most widely used anaesthetic agents (including propofol, sevoflurane, and isoflurane) potentiate GABA-mediated inhibition, which alters activity in networks spanning brainstem, thalamic, and cortical regions (Brown et al., 2010). However, whether these network changes, and concomitant transitions into and out of consciousness, are mediated by top-down (direct modulation of cortical and thalamocortical circuits) or bottom-up (ascending sub-cortical arousal systems exerting influence over cortex) processes remains contentious (Mashour and Hudetz, 2017). There are a host of highly conserved brainstem, midbrain, and forebrain nuclei whose widespread innervation exerts neuromodulatory control over the rest of the brain (Avery and Krichmar, 2017; Marder, 2012; Shine et al., 2019). These can modulate the gain of receptive neuronal populations through altering their electrical and synaptic properties, thus also affecting subsequent downstream inter-regional communication (Aston-Jones and Cohen, 2005). Moreover, these systems can act in concert to produce an adaptive system that shapes network topologies and dynamics (Brezina, 2010). Functional integrity of the default mode network (DMN), which is associated with autonoetic consciousness (Guldenmund et al., 2017; Liu et al., 2015), is reportedly under the neuromodulatory influence of dopaminergic FC during propofol anaesthesia (Spindler et al., 2021). Conversely, another recent study highlighted the importance of direct action of propofol on cortical regions, with networks showing reduced connectivity under anaesthesia also highly expressing parvalbumin positive GABAergic neurones (Craig et al., 2021). As such, both neuromodulatory and cortico-centric mechanisms seem to play a role (Lee and Mashour, 2018; Nguyen and Postnova, 2021), with a paucity of studies examining these effects in combination. Crucially, these need not be mutually exclusive, and it has been suggested that bottom-up and top-down mechanisms modulate separable dimensions of consciousness (Mashour and Hudetz, 2017).

Comprehensive accounts of brain function must integrate micro-, meso‑ and macro-scale mechanisms across different neural states (Bassett and Sporns, 2017). Conventional rest, task, and naturalistic fMRI analyses are inherently incapable of providing insights into the molecular underpinnings of the BOLD signal. This limits theoretical understanding by leaving a conceptual void between receptor level mechanisms and systems level dynamics. One solution to this has been to incorporate molecular information from positron emission tomography (PET) and single-photon emission computerized tomography (SPECT) into fMRI analyses, as in Receptor-Enriched Analysis of functional connectivity by targets (REACT), to help bridge the gap between these micro- and macro-scale systems (O. Dipasquale et al., 2019). The resultant receptor-enriched networks have demonstrated alterations under pharmacological challenge (Dipasquale et al., 2020; O. 2019; Lawn et al., 2022a) and within disease states (Cercignani et al., 2021; Martins et al., 2022; Wong et al., 2022), but have also offered a promising tool to probe the molecular substrates of consciousness and cognition. Each modulatory system engages with a set of target receptors which exhibit diversity in their patterns of expression as well as downstream effects which interact in a complex pleiotropic manner. However, each system also has transporters, engaged in movement of neurotransmitters across vesicular and synaptic membranes, which serve as a coarse grain marker for innervation and influence over a given brain region. Moreover, the widespread arborisation of projections from these small nuclei produces a spatiotemporal influence over the BOLD signal that lends itself well to REACT. As such, these transporters offer a powerful means by which to derive distinct molecular networks associated with each system that can provide new insights into the effects of anaesthesia on the brain, both at rest and during sensory stimulation.

Here, we aimed to map the functional changes induced by propofol sedation to their molecular substrates, identifying neurotransmitters which may play a fundamental role in shaping the characteristic network changes seen during anaesthesia. We explored the molecular-enriched functional architecture of the brain and its changes under anaesthesia, both at rest and during a naturalistic listening condition, hypothesising that propofol would produce divergent effects on connectivity of neuromodulatory systems at rest and during naturalistic stimulation. Specifically, we performed secondary analyses of an openly available fMRI dataset of healthy subjects collected at rest and whilst listening to an emotionally engaging story. Both conditions were acquired under four states of consciousness, dependant on the level of propofol administered: awake, light sedation, deep sedation, and recovery. We derived molecular-enriched networks associated with the transporters of the main modulatory neurotransmitter systems, namely noradrenaline (NAT), dopamine (DAT), serotonin (SERT), and vesicular acetylcholine (VAChT), as well as the ionotropic GABA-A receptor. These systems reflect bottom-up neuromodulatory as well as predominantly cortical primary pharmacological mechanisms respectively. We then assessed which of these networks demonstrate functional changes induced by the different levels of propofol sedation; examined if these networks are significantly reshaped by the highly engaging external auditory drive; and tested if these conditions (rest and naturalistic stimulation) are differentially affected at varying levels of consciousness.

## 2 Methods

### 2.1 Participants

In this work, we employ data from previously published studies (Kandeepan et al., 2020; Naci et al., 2018) made publicly available on the OpenNeuro data repository (doi:10.18112/openneuro.ds003171.v2.0.0). This includes data from 17 healthy subjects (Age: 24± 5, M/F: 13/4) who were right-handed, native English speakers, and showed no history of neurological disorders. The original study gained full ethical approval from the Health Sciences Research Ethics Board and Psychology Research Ethics Board of Western University (REB #104,755) and was conducted in accordance with the revised declaration of Helsinki (2000).

### 2.2 Study design

Participants underwent an fMRI scan whilst listening to an audio clip and then at rest during four sequential levels of consciousness: awake, light sedation, deep sedation, and recovery. The audio clip was a 5-minute excerpt from the movie “Taken” depicting a teenage girl being kidnapped, intended to be highly emotionally evocative and arousing. Both story and resting-state runs were conducted with closed eyes for all states of consciousness.

### 2.3 Anaesthesia

The different states of consciousness were defined as 1) Awake: Prior to propofol administration, participants were fully awake, alert, and communicative. 2) Light sedation: propofol infusion commenced with a target effect-site concentration of 0.6 µg/ml and oxygen titrated to maintain SpO2 above 96%. Once the baseline target effect-site concentration was achieved, participants’ level of sedation was assessed. Propofol produced increased calmness and slowed verbal responsiveness. Participants were considered lightly anaesthetised (Ramsey level 3) when they stopped engaging in spontaneous conversation, speech became sluggish, and only responded to loud commands. Once achieved, the effect-site concentration was maintained. 3) Deep sedation: the target effect-site concentration was further increased in increments of 0.3 µg/ml with repeated assessments of responsiveness. Participants were considered deeply sedated (Ramsey level 5) when they stopped responding to verbal commands, were unable to converse, and were only rousable to light physical stimulation. Once reached, the participant was maintained at that level. Participants remained capable of spontaneous cardiovascular function and ventilation. 4) Recovery: following the deep sedation runs, propofol administration was discontinued and approximately 11 min later participants reached Ramsey level 2, with clear and quick response to verbal commands.

### 2.4 MRI acquisition

Participants were provided with noise cancelling headphones (Sensimetrics, S14; www.sens.com) to deliver sound at an individualised volume deemed comfortable. Imaging was performed on a 3T Siemens Tim Trio system with a 32-channel head coil. Subjects underwent audio and resting state fMRI scans using the same BOLD EPI sequence for both conditions (33 slices, voxel size: 3mm^3^ isotropic, inter-slice gap of 25%, TR = 2000 ms, TE = 30 ms, matrix size = 64×64, FA = 75°). The audio and resting-state scans had 155 and 256 vol respectively. Anatomical scans were also obtained using a T1-weighted 3D Magnetization Prepared - Rapid Gradient Echo (MPRAGE) sequence (voxel size: 1mm^3^ isotropic, TR = 2.3, TE = 4.25 ms, matrix size = 240 × 256 × 192, FA = 9°).

### 2.5 Image pre-processing

Data were pre-processed using FMRIB Software Library (FSL) (https://fsl.fmrib.ox.ac.uk/fsl/fslwiki/). The processing steps included volume re-alignment with MCFLIRT (Jenkinson et al., 2002), non-brain tissue removal utilising the brain extraction tool (BET)(Smith, 2002), spatial smoothing with a 6 mm FWHM Gaussian Kernel, and denoising utilising the Independent Components Analysis-based Automatic Removal Of Motion Artefacts (ICA-AROMA)(Pruim et al., 2015). Furthermore, subject-specific white matter (WM) and cerebrospinal fluid (CSF) masks were generated from segmentation of structural images, eroded to reduce partial volume effects with grey matter (GM), co-registered to the subject-specific functional space, and used to extract and regress out mean WM and CSF signals from each participant's functional image time-series. Finally, data were high-pass temporal filtered with a cut off frequency of 0.005 Hz, normalised to the standard MNI152 template space, and resampled at 2 mm^3^ resolution.

### 2.6 Population-based molecular templates

**Fig. 1:**
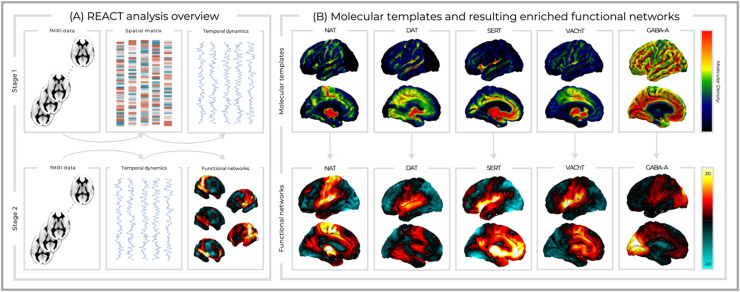
The REACT analysis framework and molecular-enriched functional networks. (A) The stage 1 general linear model (GLM) uses the vectorised PET maps as a spatial design matrix to extract the dominant BOLD fluctuations within each molecular system. The second GLM regresses these against the time series from each voxel to generate receptor-enriched maps of FC. (B) The different PET templates employed within the REACT analysis (upper row) as well as the resultant molecular-enriched networks (lower row) which are shown averaged across participants, states, and conditions (see SI fig-2) for separate figures of each combination of condition and state.

We employed transporter and receptor density maps from the noradrenergic, dopaminergic, serotonergic, cholinergic, and GABAergic systems (Fig. 1). The NAT template was derived from 10 healthy individuals utilising S, S-[^11^C]O-methylreboxetine PET (Hesse et al., 2017). DAT is from a publicly available template of 123I-Ioflupane single-photon emission computerized tomography (SPECT) images from 30 healthy subjects (HS) without evidence of nigrostriatal degeneration (https://www.nitrc.org/projects/spmtemplates)(García-Gómez et al., 2013). The SERT map was derived from the [^11^C]DASB PET images of 16 healthy controls (internal PET database). ^18^F-fluoroethoxybenzovesamicol PET was used to produce the VAChT template from 12 healthy participants (Aghourian et al., 2017). The GABA-A template was derived from 6 healthy individuals utilising [^11^C]flumazenil ((Myers et al., 2012) as described in (Dukart et al., 2018)). For each template, voxels within regions used as a reference for quantification of the molecular data in the kinetic model were replaced with the minimum value across all GM voxels, in order to minimise the contribution of those regions without excluding them from the main analysis (occipital cortex for NAT and DAT as well as cerebellum for SERT and VAChT). Finally, all templates were normalised by scaling image values between 0 and 1 whilst preserving the intensity distribution. To examine collinearity between the receptor systems, we calculated the correlation coefficients between each pair of PET templates (SI fig-1) as well as their Variance Inflation Factors (VIF). Of note, VIF quantifies the severity of multicollinearity in an ordinary least squares regression analysis ($\text{VIF}=\frac{1}{1\;-\;R^{2}}$), with higher values (i.e., above 5) denoting strong collinearity.

### 2.7 Receptor-enriched analysis of functional connectivity

The functional networks enriched by the molecular systems (NAT, DAT, SERT, VAChT, and GABA-A) were estimated for each condition (audio and rest) and state (awake, light sedation, deep sedation, and recovery) using a two-step multiple linear regression analysis (O. Dipasquale et al., 2019) implemented in the REACT toolbox (https://github.com/ottaviadipasquale/react-fmri (Dipasquale and Frigo, 2021)). An overview of the REACT analysis is reported in Fig. 1A. The molecular templates were used in the first regression analysis as spatial regressors to estimate the dominant BOLD fluctuation of the functional network related to each molecular system. At this stage, both the fMRI data and the design matrix (i.e., the molecular templates) were demeaned and masked using a binarized GM atlas derived from all the molecular data, to restrict the analysis to only those GM voxels for which receptor density information was available. The resulting subject-specific time series were then used as temporal regressors in the second multiple regression analysis, to estimate the subject-specific target-enriched functional maps. This second step was restricted to a binarized GM masque derived from all participants. Again, both data and design matrix (i.e., the time series estimated in the first step) were demeaned, with the latter also being normalised to unit standard deviation. The receptor-enriched network maps were averaged across participants, conditions and states for visualisation purposes, but also for each condition and state separately to provide a detailed view of these systems. The networks derived at rest in the awake state were also further anatomically contextualised by calculating the probability of them including each region in the Harvard-Oxford cortical and sub-cortical atlases (SI fig-3). These values were determined by thresholding the mean molecular-enriched FC maps arbitrarily at 3 to derive the rough network configuration (these images were not used for any statistical inference) before using the FSL “atlasquery” command to anatomically label regions belonging to each network.

### 2.8 Statistical analysis

A repeated measures ANOVA was implemented within the Multivariate and Repeated Measures (MRM) toolbox (McFarquhar et al., 2016) to compare networks across conditions and states. For each receptor system, we ran a 2 × 4 repeated measures ANOVA with the within-subject factors Condition (audio/rest) and State (awake/light sedation/deep sedation/recovery). Each model used 5000 permutations and cluster-based thresholding (cluster-forming threshold *p* = 0.001). Results were family wise error (FWE) corrected for multiple comparisons as well as Bonferroni corrected across receptor systems (*p* < 0.05 / 5). Mean receptor-enriched FC was extracted from significant clusters for each participant in each condition and state. These summary estimates were used to compute lower-level post-hoc pairwise tests (Sidak corrected for multiple comparisons) within SPSS (version 28) in order to determine which conditions and states were driving the significant ANOVA results.

## 3 Results

The VIF values for the receptor maps were 1.80, 2.27, 3.56, 3.72, and 1.06 for NAT, DAT, SERT, VAChT, and GABA-A respectively, reflecting a low-moderate level of collinearity and confirming their suitability for inclusion together within the multiple linear regression REACT analysis. This delineated receptor-enriched FC maps associated with each neurotransmitter system for each participant, state, and condition. Averaged across participants, these molecular-enriched functional systems showed overlapping yet distinct patterns of connectivity between receptor systems (Fig. 1B) as well as some degree of modification across conditions and states (SI fig-2). The networks from the resting awake run were further contextualised through quantifying the probability of each anatomical region being included within each molecular-enriched system (SI fig-3). Positive receptor-enriched FC of varying strength was seen within similar bilateral temporal, opercular, and insular regions across all the modulatory systems, highlighting a potential focal region for neuromodulatory molecular-enriched networks. The SERT network showed additional hubs within the frontal pole, anterior cingulate, and paracingulate cortex. NAT-enriched FC was particularly strong within the pre- and post-central gyri. Both the DAT and VAChT networks showed strong connectivity within the caudate and putamen, though the former was stronger, within the lingual gyrus, precuneus, and brainstem whilst the latter was stronger within the anterior cingulate, paracingulate, and thalamus. Finally, the GABA-A network showed primary hubs within the occipital pole and lingual gyrus, but also the intracalcarine, lateral occipital, cuneus, and precuneus cortex. For full details, see SI fig-3.

### 3.1 Interaction of condition and state

**Fig. 2:**
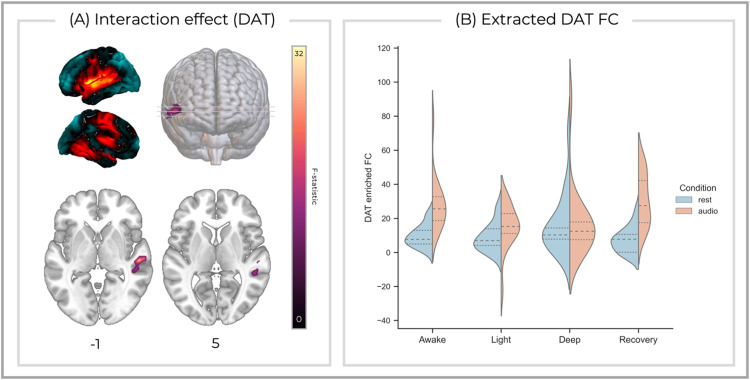
(A) DAT-enriched FC within the right middle/superior temporal gyrus and mid-cingulate demonstrated an interaction between conditions and states that remained significant after Bonferroni correction for multiple comparisons across systems. The average molecular enriched networks are shown on the top left and *z* MNI co-ordinates are reported below each axial slice. (B) Mean DAT enriched FC extracted from the significant cluster displayed for each conditon and state. Dotted lines represent quartiles. Image slices are shown in the neurological orientation.

We found an interaction effect in the DAT-enriched network (Fig. 2), with significant clusters surviving Bonferroni correction located in the right middle/superior temporal gyrus (F(3,48) = 32.1, *p* < 0.001, cluster size = 235, peak MNI [*x* = 54, *y* = −20, *z* = 0]). Simple main effects analyses revealed no significant differences during the rest condition. However, during the auditory condition, FC was significantly greater during the awake state compared to light (mean difference = 13.1, SE = 4.90, *p* = 0.019, CI = 2.74 to 23.5) and deep (mean difference = 11.1, SE = 3.20, *p* = 0.003, CI = 4.31 to 17.9) sedation. Similarly, FC during the recovery state was greater than during light (mean difference = 15.7, SE = 5.22, *p* = 0.048, CI = 4.68 to 26.8) and deep sedation (mean difference = 13.7, SE = 4.54, *p* = 0.047, CI = 4.12 to 23.4). No significant interaction effects were found in the other molecular-enriched networks.

### 3.2 Main effect of state

**Fig. 3:**
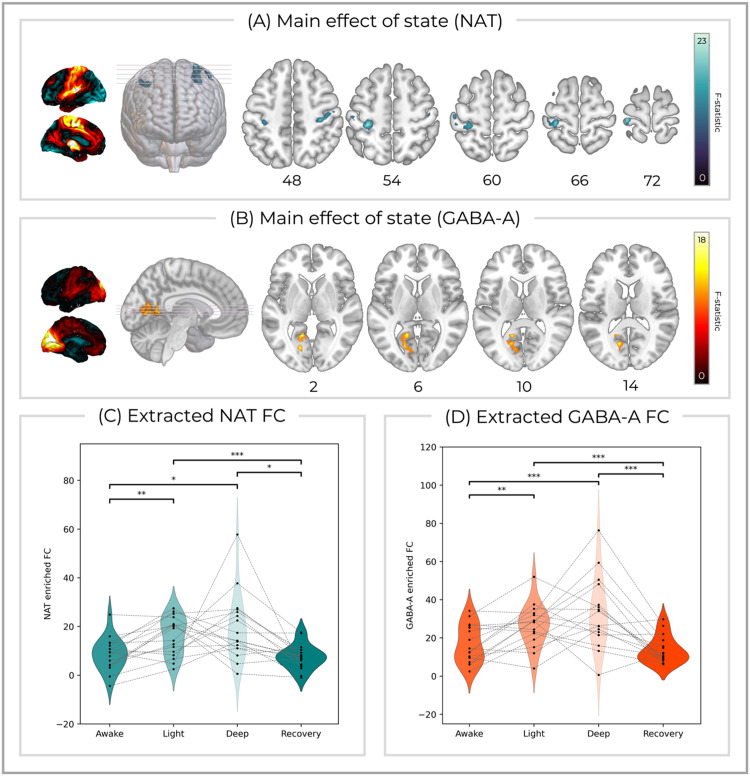
Clusters showing main effects of state (awake/light sedation/deep sedation/recovery) for (A) NAT-enriched FC and (B) GABA-A enriched FC that survived Bonferroni correction for multiple comparisons across systems. The average molecular enriched networks are shown on the left and *z* MNI co-ordinates are reported below each axial slice. The receptor-enriched FC extracted from the significant (C) NAT and (D) GABA-A clusters, averaged across conditions, showed significant differences in the post-hoc contrasts [awake < light sedation], [awake < deep sedation], [light sedation > recovery], and [deep sedation > recovery]. (*** *p* < 0.001; ** *p* < 0.01, * p < 0.05). Image slices are shown in the neurological orientation.

Altered states of consciousness were associated with changes in NAT-enriched FC within bilateral primary somatosensory cortex (F(3,48) = 23.4, *p* = 0.001, cluster size = 319, peak MNI [*x* = 34, *y* = −24, *z* = 46]) (Fig. 3A/C). The post-hoc test revealed that, compared to the awake state, FC was significantly greater during light (mean difference = 7.70, SE = 1.80, *p* = 0.004, CI = 3.86 to 11.5) and deep sedation (mean difference = 10.9, SE = 3.36, *p* = 0.029, CI = 3.81 to 18.0). Additionally, FC was significantly reduced during the recovery state compared to light (mean difference = 8.67, SE = 1.70, *p* < 0.001, CI = 5.07 to 12.3) and deep sedation (mean difference = 11.9, SE = 3.33, *p* = 0.015, CI = 4.87 to 19.0). Similarly, GABA-A-enriched FC showed a main effect of sedation within the left lingual gyrus, precuneus, cuneus, and posterior cingulate (F(3,48) = 18.5, *p* = 0.002, cluster size = 264, peak MNI [*x* = −14, *y* = −64, *z* = 8])( Fig. 3B/D). This was also driven by increased FC during the light (mean difference = 9.75, SE = 2.60, *p* = 0.011, CI = 4.23 to 15.3) and deep sedation states (mean difference = 16.5, SE = 3.35, *p* < 0.001, CI = 9.40 to 23.6), compared to when participants were awake as well as reductions in FC during the recovery state compared to light (mean difference = 12.2, SE = 2.72, *p* = 0.002*,* CI = 6.44 to 18.0) and deep sedation (mean difference = 19.0, SE = 4.19, *p* = 0.002, CI = 10.1 to 27.9). No other networks showed a significant main effect of State.

### 3.3 Main effect of condition

**Fig. 4:**
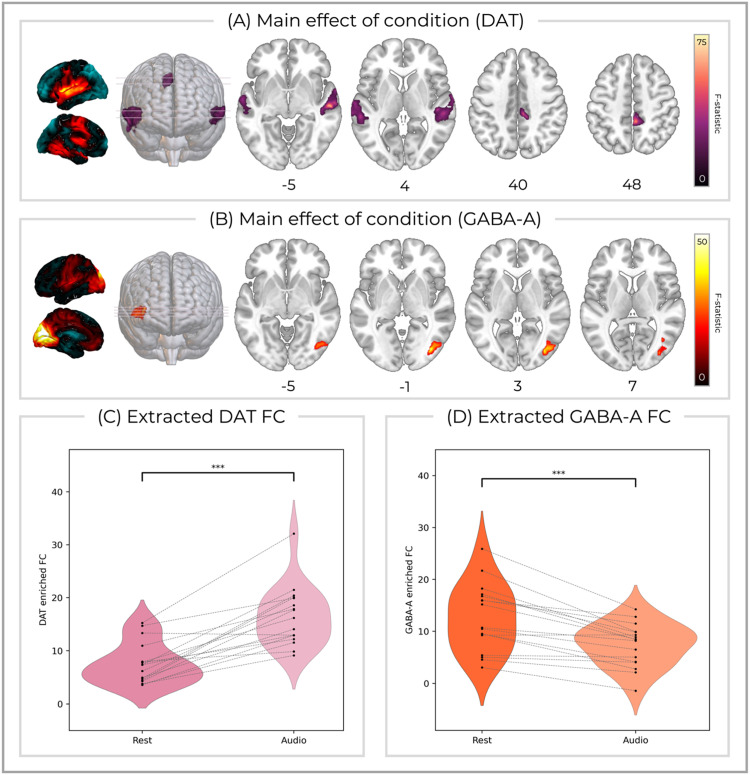
(A) DAT-enriched FC within bilateral middle/superior temporal gyrus and mid-cingulate/precuneus as well as GABA-A enriched FC within right lateral occipital cortex showed a main effect of condition (rest/audio) that remained significant after Bonferroni correction for multiple comparisons across systems. The average molecular enriched networks are shown on the left and *z* MNI co-ordinates are reported below each axial slice. Mean (C) DAT and (D) GABA-A enriched FC extracted from the significant clusters averaged across states, and displayed between conditions for each participant. Image slices are shown in the neurological orientation.

A significant difference between conditions of rest and auditory stimulation was found for DAT-enriched FC, located in right middle/superior temporal gyrus (F(1,16) = 62.4, *p* = 0.000, cluster size = 1110, peak MNI [*x* = 58, *y* = −19, *z* = −4]), left middle/superior temporal gyrus (F(1,16) = 76.7, *p* = 0.001, cluster size = 779, peak MNI [*x* = −61, *y* = −13, *z* = −1]), as well as right mid-cingulate and precuneus (F(1,16) = 65.1, *p* = 0.004, cluster size = 329, peak MNI [*x* = 5, *y* = −40, *z* = 50] (Fig. 4A). The post-hoc comparison revealed reduced DAT-enriched FC during the auditory condition compared to rest (mean difference = 8.91, SE = 1.23, *p* < 0.001, CI = 6.30 to 11.5)(Fig. 4C). GABA-enriched-FC also showed a significant main effect of condition in the right lateral occipital cortex (F(1,16) = 49.4, *p* = 0.008, cluster size = 302, peak MNI [*x* =, *y* = −76, *z* = −2])(Fig. 4B), with the post-hoc test demonstrating reduced GABA-enriched FC during the auditory condition compared to rest (mean difference = 5.26, SE = 0.88, *p* < 0.001, CI = 3.39 to 7.14)(Fig. 4D). Finally, VAChT-enriched FC within the right superior temporal gyrus showed a significant main effect of condition, but this did not survive Bonferroni correction for multiple comparisons across systems. No significant main effects of Condition were found in the other functional networks.

## 4 Discussion

In this work, we explored how different levels of propofol sedation shape the network architecture of the resting and engaged brain, utilising novel multi-modal methods which establish clearer mechanistic links between neurotransmission and connectivity. We enriched BOLD fMRI analysis with the distribution density of modulatory (NAT, DAT, SERT, and VAChT) as well as inhibitory (GABA-A) neurotransmitters and assessed the connectivity of these networks under propofol-mediated manipulations of consciousness. Given the potential benefit of using naturalistic stimuli to engage sensory and higher-level cognitive processes (Finn, 2021), we also tested if these molecular-enriched functional networks undergo substantial reconfiguration as compared to the resting state. We found a significant modification of the DAT-enriched network under external auditory drive, mainly within bilateral temporal regions and the mid-cingulate/precuneus, with the right temporal gyrus demonstrating differential effects as a function of consciousness. Moreover, propofol sedation was associated with increased GABA-A and NAT enriched FC within occipital and somatosensory regions respectively. Finally, GABA-A enriched FC within lateral occipital cortex was also reduced in the naturalistic condition. We discuss these main findings below.

### 4.1 Propofol sedation induces a differential reconfiguration of the DAT-enriched functional network during naturalistic stimulation as compared to rest

Previous work largely demonstrates that, during the awake state, auditory stimulation produces significant activations in bilateral temporal and frontal regions, of which the former but not the latter show some level of preservation under propofol sedation (Adapa et al., 2014; Heinke et al., 2004; Liu et al., 2012; Plourde et al., 2006). This would suggest that some aspects of basic sensory processing persist, but higher-level mechanisms functionally and causally downstream still preclude the integration of lower-level information into a coherent percept (Dehaene et al., 2014, 2006; Dehaene and Changeux, 2011). Somewhat contrary to this, we find that DAT-enriched FC within the right middle/superior temporal gyrus is differentially engaged by auditory processing at different levels of anaesthesia, providing indirect evidence for a role of dopamine in processing of auditory stimuli which is at least partially extinguished under anaesthesia. This somewhat aligns with the previously described role of dopamine in modulating DMN connectivity under propofol anaesthesia (Spindler et al., 2021). Although our findings are condition specific, both sets of results point towards propofol shaping network dynamics through engagement of dopaminergic circuits. Dopamine has broadly been linked to network dynamics, including linear and non-linear effects on different resting-state networks (Cole et al., 2013), modulation of network stability and integrity (Shafiei et al., 2019), the connectivity of striatal regions (Kelly et al., 2009), and distinct contributions of dopaminergic nuclei to shaping different networks (Conio et al., 2019; Murty et al., 2014). Here, we extend these accounts to suggest that the neuromodulatory role of dopamine engaged under naturalistic conditions might contribute to consciousness related perceptual and/or cognitive processes.

Dopaminergic neurotransmission over varying time scales has long been implicated in the inter-related mechanisms of action, learning, and reward processing (Berke, 2018; Diederen and Fletcher, 2021; Gershman and Uchida, 2019; Lerner et al., 2021; Schultz, 2007). Perception and action are inextricably intertwined, and a multitude of parallel systems linking perceptual processing anchored within early sensory cortices through to behavioural responses, for which the basal ganglia play a critical role (Ding and Gold, 2013; Guo et al., 2018). Indeed, a dopaminergic recipient portion of the posterior dorsal striatum is explicitly involved in processing of auditory information and shaping the selection of appropriate actions (Chen et al., 2022; L. 2019; Hunnicutt et al., 2014; Ponvert and Jaramillo, 2019; Valjent and Gangarossa, 2021; Xiong et al., 2015a, 2015b; Znamenskiy and Zador, 2013). Dopamine has also been suggested to play a key role in gating prefrontal output, akin to striatal gaiting of actions, but applied to facets of cognition such as working memory and executive function (Frank et al., 2001; Hazy et al., 2007). This top-down flexible cognitive control can bias information processing towards task-relevant representations (Cools, 2019; van Schouwenburg et al., 2015, 2013, 2010). Similarly, dopamine is indirectly implicated in aspects of attention and executive function through its role in the aetiology of attention deficit hyperactive disorder, as evidenced by genetics (Kanarik et al., 2022; Li et al., 2006; Wu et al., 2012) and the largely dopaminergic pharmacology of effective treatments (Faraone, 2018). Given this multiplicity of dopaminergic mechanisms, we conjecture that the differentially engaged DAT-enriched connectivity identified here may reflect aspects of cognition including attention or action selection, neither of which would be expected to persist under anaesthesia. The right lateralisation of this interaction effect is likely a statistical artefact resulting from the application of statistical thresholds, i.e. only one of the hemispheres survive correction even though the signal is present bilaterally. A better powered analysis would likely reveal more widespread results encompassing the additional clusters engaged during auditory stimulation. Indeed, the FC of the ventral tegmental area is largely symmetrical (Cauzzo et al., 2022), suggesting that if not solely due to sensitivity, this laterality could be driven by the temporal cortex itself, possibly due to additional mechanisms such as attentional networks which show a right hemispheric dominance (Corbetta and Shulman, 2002; Coull, 1998). Careful subsequent experimental and/or pharmacological manipulation will be required to further dissect the potential functional contributions of dopamine, and concomitant network reconfigurations, to both cognition and consciousness.

### 4.2 NAT and GABA-A enriched FC increase under propofol sedation

Consciousness appears to be dependant on the correlation and anti-correlation of large-scale brain networks (Dehaene et al., 2014; Dehaene and Changeux, 2011; di Perri et al., 2016). Increasing evidence supports the notion that these networks not only depend on their stable structural connectivity, but are also shaped by a host of neuromodulatory systems that exert widespread influence over diverse but overlapping cortical and subcortical regions (van den Brink et al., 2019). Our findings expand these accounts by showing that FC increases with depth of sedation for NAT and GABA-A enriched networks within somatosensory and occipital regions, respectively. Whilst located within regions of sensory cortex, these clusters represent coupling to a diverse set of additional brain regions highly expressing the relevant receptor/transporter, alluding to a more widespread network change which likely involves additional higher-order regions. Broadly, this aligns with the known actions of propofol directly onto GABAergic cortical circuits as well as via ascending modulatory arousal systems.

Propofol acts primarily through potentiating GABAergic transmission throughout the central nervous system (Bai et al., 1999; Hapfelmeier et al., 2001; Hemmings et al., 2019, 2005). Despite a significant number of neuroimaging studies, a comprehensive account of the relationships between this GABAergic potentiation, neural activity/connectivity, and consciousness remains elusive (Bonhomme et al., 2019). Even in the first PET studies on anaesthesia in humans, Alkire and colleagues speculated that regional reductions in glucose metabolic rates (which was greater within cortical than subcortical regions) may be driven by the distribution of GABA-A receptors (Alkire et al., 1995). Accordingly, we found that propofol sedation increased FC in the functional network related to GABA-A within occipital regions, which likely reflects direct actions of propofol on cortical GABA-A receptors modulating the BOLD activity of those areas. This is in accordance with recent whole brain modelling work which demonstrated a key role of GABA-A-mediated inhibition in recapitulating the experimentally observed network dynamics under propofol anaesthesia (Luppi et al., 2022). Similarly, another study exploiting genomic data from the Allen Human Brain Atlas (AHBA) found that networks with significantly reduced connectivity under propofol also show a high density of parvalbumin-expressing GABAergic neurones (Craig et al., 2021). Their complementary approach provides cellular meso‑scale insight, further linking global connectivity measures to the GABAergic system by particularly implicating this subpopulation of inhibitory neurones. Future work examining both receptor and cellular systems in combination may allow for more comprehensive mapping of the functional contributions of these lower-level organisational principles onto the systems-levels dynamics supporting consciousness.

Interestingly, Craig et al. also found that many areas, including the somatosensory cortex, exhibited increases in connectivity as a function of depth of anaesthesia (Craig et al., 2021). Unlike the regions of reduced connectivity mentioned above, these increases were not significantly associated with GABAergic expression, alluding to an alternative mechanism. Our results demonstrate that noradrenergic FC is increased in the bilateral somatosensory cortices during propofol sedation, suggesting that altered noradrenergic tone may contribute to these additional changes in connectivity. Propofol has been reported to supress activity within brainstem nuclei relating to arousal (Nguyen and Postnova, 2021), including the locus coeruleus (LC)(Chen et al., 1999; Du et al., 2018). The LC provides widespread noradrenergic projections to virtually all regions of the brain (Sara, 2009). As such, our findings may reflect suppression of LC activity by propofol, resulting in the selective modulation of BOLD signal in noradrenaline recipient regions. Furthermore, neuromodulators including noradrenaline also tune thalamocortical network synchronisation (Dahl et al., 2022), which has been strongly implicated in mechanisms of anaesthesia (Malekmohammadi et al., 2019). This is also concordant with various supporting evidence for a causal contribution of noradrenaline to maintaining consciousness. Dexmedetomidine, which inhibits noradrenergic neurones within the LC through presynaptic α2 adrenoceptor agonism (Nelson et al., 2003), produces a state similar to non-REM sleep and reduces the dose of propofol required to induce loss of consciousness (Peden et al., 2001; Zhang et al., 2021). Similarly, chemogenetic activation of NA populations within the LC can retard anaesthetic induction as well as produce cortical arousal and expedited behavioural emergence from unconsciousness following isoflurane anaesthesia in a manner preventable by ɑ1 or ß receptor antagonism (Vazey and Aston-Jones, 2014). Furthermore, mutations perturbing NA biosynthesis can produce hypersensitivity to anaesthetic induction and particularly diminish emergence from anaesthesia (Hu et al., 2012). However, neither pharmacological blockade of noradrenergic reuptake (Kenny et al., 2015), nor microdialysis of noradrenaline within the prefrontal cortex (Pal et al., 2018) restores consciousness during continuous sevoflurane anaesthesia. Moreover, manipulations of other modulatory neurotransmitter systems including acetylcholine, dopamine, histamine, and orexin can also modulate the neurophysiological induction, maintenance, and emergence from anaesthesia (Hemmings et al., 2019). Thus, whilst noradrenergic transmission from the LC is clearly involved, its alteration under anaesthesia seems to be neither necessary nor sufficient for the resultant behavioural manifestation. This further highlights the complex contribution of numerous mechanisms which interact at multiple levels to enact general anaesthetic agents’ effects on consciousness.

It remains unclear why we did not identify differences in the other modulatory receptor-enriched networks under propofol sedation regardless of condition. In particular, projections from the dopaminergic ventral tegmental area (VTA) to the posterior cingulate cortex (PCC) and precuneus have recently been described to modulate DMN connectivity under propofol anaesthesia (Spindler et al., 2021). The authors also attempted to demonstrate a more direct causal role for dopamine in this mechanism by showing methylphenidate boosts VTA – PCC/precuneus connectivity in patients with disorders of consciousness. However, the temporal dynamics of the VTA and LC are positively correlated, with the collective activity of brainstem nuclei generally showing widespread anticorrelation with the cortex (Zhang et al., 2016). Moreover, methylphenidate also increases levels of noradrenaline, and the LC has recently been robustly demonstrated to have the capacity to modulate frontal DMN regions (Oyarzabal et al., 2022). As such, disentangling the effects of these catecholaminergic systems on brain-wide connectivity is challenging and the aforementioned findings may be driven at least in part by the noradrenergic system. Indeed, both the PCC and precuneus showed similar levels of dopaminergic and noradrenergic transporter-enriched FC (SI fig-3). REACT allowed us to examine both DAT and NAT related FC simultaneously, and the distribution of their receptor sub-systems has previously been employed to attempt to delineate their associations to distinct patterns of connectivity induced by atomoxetine (van den Brink et al., 2018). These differences between molecular-enriched networks and seed-based connectivity approaches may simply be methodological, with our dopaminergic task-positive findings being the inverse of its effects on the task negative DMN connectivity (Spindler et al., 2021). Future work employing both methods as well as more selective manipulation of catecholaminergic transmission may allow for more precise characterisation of their relative contributions to network changes under anaesthesia.

### 4.3 DAT and GABA-A enriched connectivity associated with naturalistic auditory stimulation, regardless of level of anaesthesia

Analysis of task-based fMRI conventionally entails convolving an event or block-related design with a haemodynamic response function and then identifying voxels whose BOLD activity shows temporal concordance with this predicted time series. Conversely, here we employed a temporally coarse-grained approach by calculating a measure of static receptor-enriched FC during naturalistic auditory stimulation and comparing this to the resting state condition. This delineated a broader set of regions of the DAT-enriched network, namely the bilateral middle/superior temporal gyri as well as the mid-cingulate cortex, that demonstrated increased FC during the auditory condition as compared to rest. In other words, the BOLD time series of these clusters was more strongly coupled to the dominant fluctuations of the functional network related to DAT during the task condition than at rest. Given that DAT is principally expressed within the basal ganglia, and that the resultant average DAT-enriched network has key nodes within bilateral striatal and temporal regions, our findings likely represent a neuromodulatory role of dopamine in shaping cortico-striatal networks. Indeed, a recent tractography study identified strong structural connectivity between the superior temporal cortex and putamen (Sitek et al., 2022). Regions of mid-, posterior-cingulate, and precuneus cortex are also innervated by dopaminergic afferents (Vogt, 2016) and have previously been described to preferentially activate during narrative shifts within a naturalistic listening paradigm (Whitney et al., 2009). As discussed above, precisely which facets of perception and/or cognition this connectivity may relate to represents remains speculative, although, we conjecture this may reflect mechanisms relating to attention or action selection. Similarly, the reduction of GABA-A enriched FC within right lateral occipital cortex during auditory stimulation could reflect a multitude of different perceptual and cognitive mechanisms. Thus, whilst this work provides a proof-of-concept that molecular-enriched networks are also amenable to reconfiguration under non-pharmacological or pathological states, more specific experimental manipulation is required to tease apart their functional significance. In particular, the combined use of this temporally coarse grained approach alongside a modified version of generalised psycho-physiological interaction analysis (Wong et al., 2022) utilising REACT molecular time series may prove a particularly fruitful approach to probe task-related molecular-enriched connectivity associated with specific facets of cognition and perception engaged under diverse naturalistic and task conditions. Moreover, investigation of the potential differential contributions of D1- and D2-like receptors, as well as the broader interactions of different modulatory systems within the focal temporal regions showing positive receptor-enriched FC across modulatory systems, will be important to further link these receptor-enriched networks to their precise roles in mediating or modulating the subjective experience associated with naturalistic auditory stimulation.

### 4.4 Limitations

This work is not without limitations. Firstly, the small sample size may limit our power to detect alterations within these receptor-enriched networks and interactions between conditions and states, especially alongside the stringent Bonferroni correction applied. Similarly, stronger doses of propofol may produce more substantial effects on molecular-enriched networks than those seen here. Secondly, the PET templates employed within the REACT analyses were average maps derived in separate cohorts of healthy individuals. Thus, accounting for inter-individual differences in receptor density in our sample is not possible. However, the use of independent average templates brings the advantage of permitting investigation of multiple targets without requiring the acquisition of multi-tracer data in the same subject, which is typically not feasible. Finally, the spatial distributions of molecular targets we studied here do show some level of correlation between each other. However, including all maps within the same model is an essential step to ensure that the variance of the BOLD signal is partitioned between all systems, instead of running separate models for each different system, which would lead to an omitted-variable bias. This bias occurs when a statistical model (here, multiple linear regression) omits an independent variable (a molecular system) that is both a determinant of the dependant variable (the BOLD signal) and correlated with one or more of the included independent variables (Lawn et al., 2022b). This yields an unpredictable attribution of the effects of the missing variables to those variables that are included. Furthermore, the VIF values of the molecular systems included in this study were all within a reasonable range (all < 5), providing additional confidence in the validity and interpretability of our models.

## 5 Conclusion

In this work, we provide new evidence that propofol engages with both cortical and sub-cortical targets to shape the network architecture of the brain during anaesthesia. Furthermore, we delineate a dopaminergic network which shows cognition-related reconfiguration and differential modulation under anaesthesia. This novel application of REACT highlights the significant potential of this methodology to further unravel the contribution of molecular systems to various facets of perception and cognition. Future work examining interactions with other transmitter systems, the contribution of receptor subtypes, and dynamic fluctuations in receptor-enriched FC may offer additional critical insights into neuromodulatory mechanisms engaged under naturalistic conditions. Furthermore, characterising receptor-enriched network changes across diverse anaesthetic agents may shed further light on the functional contributions of these molecular systems as well as aid identification of common paths to unconsciousness. In the longer term, an understanding of how molecular mechanisms shape the complex systems level dynamics from which consciousness emerges may offer novel opportunities to treat those suffering from disorders of consciousness.

## Data availability statement

The data is fully available on the Open Neuro data repository (doi: 10.18112/openneuro.ds003171.v2.0.0).

## Code availability statement

The code for the Receptor-Enriched Analysis of functional Connectivity by Targets (REACT) analysis has been made fully available as a Python software package (https://github.com/ottaviadipasquale/react-fmri)

## Data Availability

Data is fully available on OpenNeuro. The code for the REACT analysis is fully available as a Python toolbox.

## Appendix Supplementary materials

Image, application 1
