# Supplementary material for: The effects of propofol anaesthesia on molecular-enriched networks during resting-state and naturalistic listening
Source: Neuroimage. 2023 May 1;271:120018. doi: 10.1016/j.neuroimage.2023.120018 (PMC10410200; doi:10.1016/j.neuroimage.2023.120018)
Supplement: Supplementary file 1 [file mmc1.pdf]

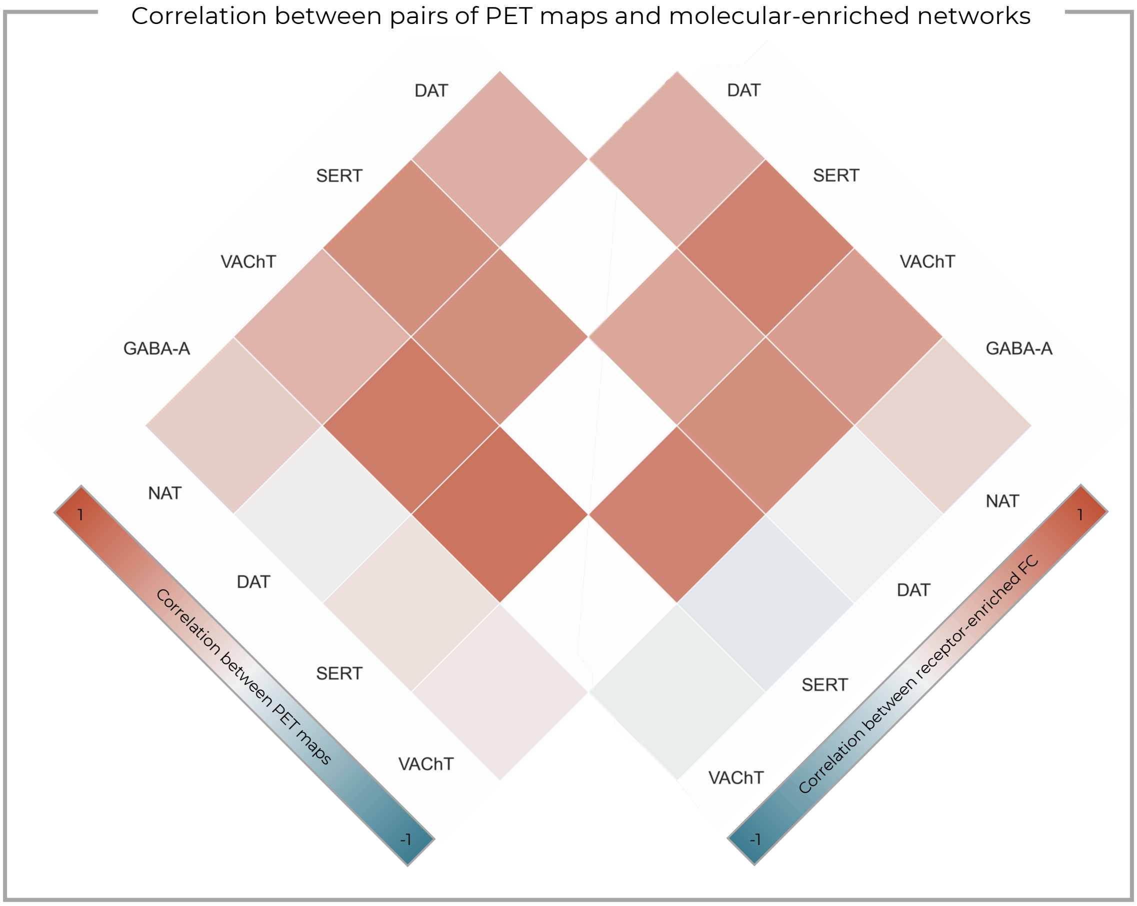

Supplementary figure 1: the correlation between each of the PET receptor/transporter maps included in the first GLM (shown on the left) as well as the correlations between the resultant molecular-enriched networks (shown on the right)

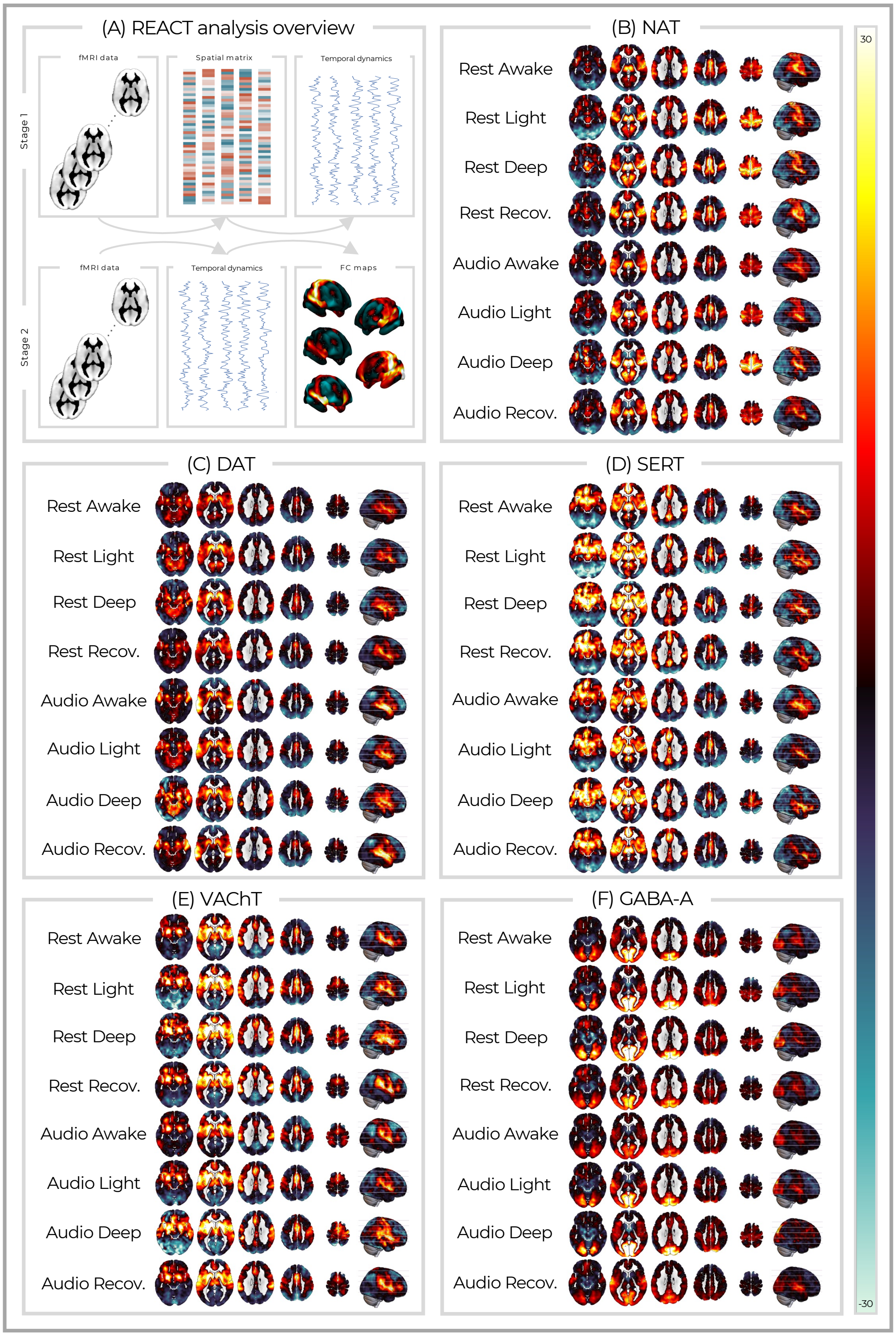

Supplementary figure 2: : (A) The REACT analysis framework and molecular-enriched functional networks(A) The stage 1 general linear model (GLM) uses the vectorised PET maps as a spatial design matrix to extracts the dominant BOLD fluctuations within each molecular system. The second GLM regresses these against the time series from each voxel to generate receptor-enriched maps of FC associated with NAT (B), DAT (C), SERT (D), VAChT (E), and and GABA-A (F). These networks are shown averaged across participants for each combination of condition (rest/audio) and state (awake/light sedation/deep sedation).

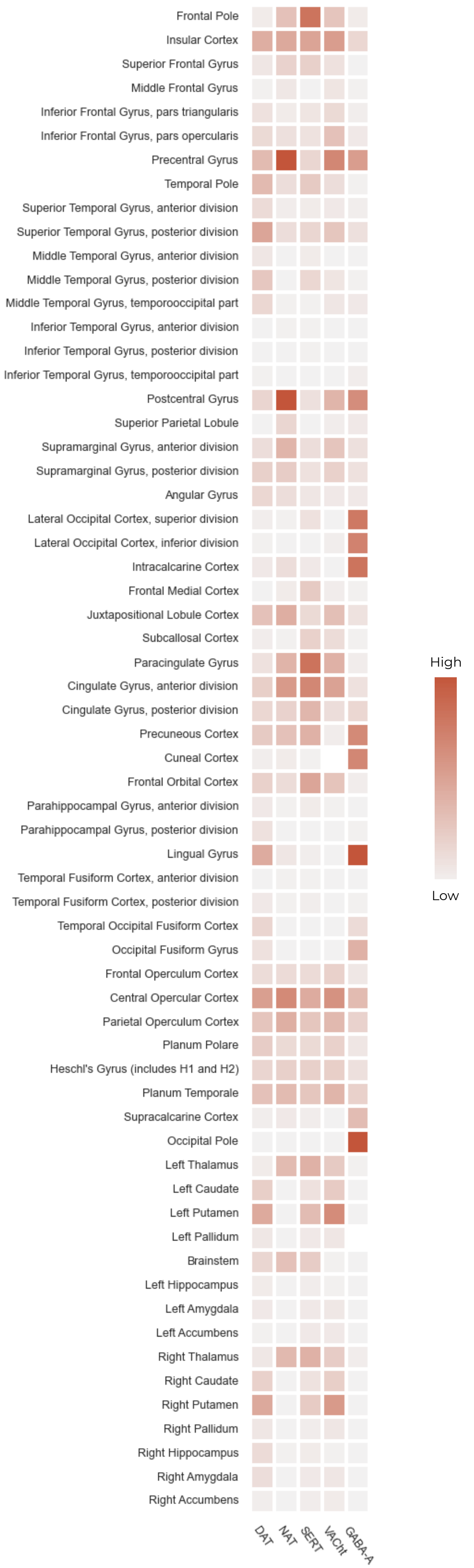

Supplementary figure 3: The probability of each anatomical region in the Harvard-Oxford cortical and sub-cortical anatomical atlases being a part of each molecular-enriched network. Values shown are only for the networks derived whilst participants were in the resting condition and awake state. These values were determined by thresholding the networks arbitrarily at 3 to derive only regions of positive FC before calling the FSL "atlasquery" command. The magnitude of the values does not convey important information, but the relative values for each network help demonstrate similarities and differences between each molecular-enriched network.
